# Insights into single-timepoint ASL hemodynamics: what visual assessment and spatial coefficient of variation can tell

**DOI:** 10.1007/s11547-024-01777-z

**Published:** 2024-02-08

**Authors:** Francesca Benedetta Pizzini, Ilaria Boscolo Galazzo, Valerio Natale, Federica Ribaldi, Max Scheffler, Ferdinando Caranci, Karl-Olof Lovblad, Gloria Menegaz, Giovanni B. Frisoni, Matthias Gunther

**Affiliations:** 1 Department of Engineering for Innovation Medicine, University of Verona, Verona, Italy; 2 Laboratory of Neuroimaging of Aging (LANVIE), University of Geneva, Geneva, Switzerland; 3 Memory Clinic, Geneva University Hospitals, Geneva, Switzerland; 4 Division of Radiology, Geneva University Hospitals, Geneva, Switzerland; 5 Department of Medicine of Precision, School of Medicine, “Luigi Vanvitelli” University of Campania, Naples, Italy; 6 Imaging Physics, Fraunhofer Institute for Digital Medicine MEVIS, Bremen, Germany; 7 Dept. of Diagnostic and Public Health, Rivoli Hospital, Rivoli, Turin, Italy

**Keywords:** Perfusion, Hemodynamics, Biomarkers, Dementia, Arterial spin labeling

## Abstract

**Purpose:**

Arterial spin labeling (ASL) represents a noninvasive perfusion biomarker, and, in the study of nonvascular disease, the use of the single-timepoint ASL technique is recommended. However, the obtained cerebral blood flow (CBF) maps may be highly influenced by delayed arterial transit time (ATT). Our aim was to assess the complexity of hemodynamic information of single-timepoint CBF maps using a new visual scale and comparing it with an ATT proxy, the “coefficient of spatial variation” (sCoV).

**Material and methods:**

Individual CBF maps were estimated in a memory clinic population (mild cognitive impairment, dementia and cognitively unimpaired controls) and classified into four levels of delayed perfusion based on a visual rating scale. Calculated measures included global/regional sCoVs and common CBF statistics, as mean, median and standard deviation. One-way ANOVA was performed to compare these measures across the four groups of delayed perfusion. Spearman correlation was used to study the association of global sCoV with clinical data and CBF statistics.

**Results:**

One hundred and forty-four participants (72 ± 7 years, 53% women) were included in the study. The proportion of maps with none, mild, moderate, and severe delayed perfusion was 15, 20, 37, and 28%, respectively. SCoV demonstrated a significant increase (*p* < 0.05) across the four groups, except when comparing none vs mild delayed perfusion groups (*p*_Bonf_ > 0.05). Global sCoV values, as an ATT proxy, ranged from 67 ± 4% (none) to 121 ± 24% (severe delayed) and were significantly associated with age and CBF statistics (*p* < 0.05).

**Conclusion:**

The impact of ATT delay in single-time CBF maps requires the use of a visual scale or sCoV in clinical or research settings.

**Supplementary Information:**

The online version contains supplementary material available at 10.1007/s11547-024-01777-z.

## Introduction

Arterial spin labeling (ASL) MRI uses magnetically labeled arterial blood water protons as an endogenous tracer for measuring tissue perfusion in a reproducible manner, without intravenous contrast agent and free of radiation [1]. For these reasons, ASL is increasingly used in clinical routine, rapidly maturing as an important tool for the assessment of different conditions, including neurodegeneration [2, 3]. Previous studies in dementia and mild cognitive impairment (MCI) have demonstrated hypoperfusion patterns remarkably similar to FDG-PET hypometabolism, with the latter still being performed more frequently in neurodegenerative disorders [3–5].

Despite the relevant information enclosed in CBF maps, these are largely affected by physiological and/or pathological individual variability in arterial transit time (ATT) [3, 6], defined as the time necessary for labeled blood to reach the microvasculature of the imaging volume [7]. This variability in ATT is more widely recognized in research focused on vascular diseases where multi-timepoint sequences are recommended [8]. Notably, this approach allows measuring several perfusion parameters, including ATT itself, while improving absolute CBF quantification and conveying more overall information on cerebrovascular health. While advantages are undeniable, its current applicability is hampered by several technical issues, in particular difficult implementation, limited volume coverage, low SNR and increased acquisition time [3, 6], all aspects that preclude the possibility to use it routinely instead of conventional single-timepoint approaches.

Moreover, ATT heterogeneity between subjects and brain regions severely impacts on CBF image quality, even in conditions where acquisition parameters and post-processing rigorously follow the recommended guidelines [3, 9]. In fact, prolonged ATT can affect images mainly causing: (1) a signal drop at the top slices (e.g., at the level of the centra semiovale), an effect partly corrected by CBF quantification method and mitigated by 3D readouts, (2) a signal drop with surrounding high intravascular signal between cerebral vascular territories (border zones, BZs or watershed areas located at the junction between two main arterial territories) which represent bilateral areas of critical blood supply, related to a combination of delayed ATT and reduced CBF or either of the two [10, 11].

In this regard, Mutsaerts et al. proposed a *quantitative* measure called spatial coefficient of variation (sCoV) that can be used as ATT proxy, extractable from single-timepoint scans without requiring additional sequences [12]. The authors demonstrated in a cohort of older patients with hypertension that sCoV predicts ATT with high precision and could aid the clinical interpretation of ASL data, especially in patients with possibly compromised cerebral vasculature, as further investigated more recently on moyamoya [13], steno-occlusive disorder [14, 15], and MCI/early dementia [7, 16, 17]. However, while a comprehensive evaluation of sCoV potential is provided in these articles, no study to date has investigated the agreement between this ATT proxy and a more practical qualitative visual assessment of individual hemodynamic patterns—which could be a useful tool in clinical practice.

This *quality control (QC)* may be a slowing step in clinical routine, yet it is indispensable to avoid underestimating the impact of ATT and recognize the presence of possible hemodynamic artifacts, mainly arterial transit artifacts (ATA). Several visual assessment scales have been proposed, dedicated however to specific pathological conditions, e.g., to aid detection of arteriovenous malformations [18], or localization of an arterial occlusion site in acute ischemic stroke [19]. In [20], a general QC scale for the clinical evaluation of ASL maps from QUASAR was proposed. This evaluates both the image contrast between anatomical structures and the presence of artifacts. This scale was not specifically developed for CBF maps, but also for other ancillary maps that can be derived from the same sequence, i.e., ATT, arterial blood volume, and T1 relaxation maps. While this approach could reasonably be applied to healthy controls or patients with a range of different diseases, it remains a complex system requiring the assessment of several contrast and artifacts’ items and therefore difficult to use in clinical practice.

Using visual *QC* or automatically derivable *quantitative* measures of hemodynamic impairment could pave the way toward a more objective interpretation of ASL images, help in equivocal cases, and provide novel insights into cerebrovascular integrity.

Therefore, the aim of our study was to assess cerebral perfusion and hemodynamic status on a large dataset of older subjects by proposing a practical visual scale and by comparing it with a quantitative measure, the sCoV. Both approaches reveal additional information hidden at first glance in single-timepoint ASL data.

## Materials and methods

A retrospective cross-sectional study was performed on a clinical population of 169 subjects (MCI, dementia, cognitively unimpaired controls, Table 1) recruited at a memory clinic from 2018 to 2020 after obtaining institutional review board approval.

**Table 1:** Study population

	Cognitively unimpaired	Mild cognitive impairment	Dementia
	(*n* = 36)	(*n* = 88)	(*n* = 20)
Sex (women)	19 (53%)	44 (50%)	13 (65%)
Age (years)	70.4 ± 7.1	72.9 ± 6.7	69.5 ± 8.6
MMSE	28.3 ± 1.1	26.3 ± 1.9	18.7 ± 4.5
ARWMC	5.8 ± 5.7	6.4 ± 4.7	7.3 ± 4.3

Figures denote number (%) or mean ± standard deviation. *ARWMC* age-related white matter changes; *MMSE* Mini–Mental State Examination

All subjects provided written consent to participate and underwent MRI scanning along with clinical, neurological and cognitive evaluation, as detailed in [21].

Twenty subjects were discarded due to MRI inconsistencies or missing clinical/demographic information, leading to a final sample of 149 individuals.

### Imaging protocol and processing

MRI was performed on a 3 T Siemens Skyra scanner (Siemens Healthineers, Germany) with a 64-channel head coil and included: (1) 3D T1 (TR/TE = 1930/2.36 ms; resolution = 0.9 mm isotropic; 208 sagittal slices); (2) 3D-GRASE FAIR ASL (TR/TE = 5000/16.38 ms; bolus duration/*T*I = 800/2000 ms; resolution = 1.5 × 1.5 × 3 mm^3^; 40 axial slices; 2 label/control pairs, 12 segments with EPI factor = 21, no vascular suppression). The ASL sequence used matched the one that was commercially available for the scanner and clinically used.

Analysis of the ASL data was performed using BASIL within FSL 6.0.3 (FMRIB Software Library). The standard mono-compartment model [22] was applied, with default relaxation values (tissue *T*1 = 1.3 s, arterial *T*1 = 1.65 s). Equilibrium blood magnetization was estimated using cerebrospinal fluid as reference. Adaptive spatial regularization on perfusion was applied [23]. CBF maps were rigidly registered to the corresponding T1-weighted images (FLIRT) and spatially normalized to the 2-mm MNI space (FNIRT).

### Raters and visual evaluation

CBF images were independently evaluated by two raters (FBP, neuroradiologist with 17 years of experience in ASL reporting; VN, neuroradiologist in training), and the individual ratings then combined to yield the final score.

**Fig. 1:**
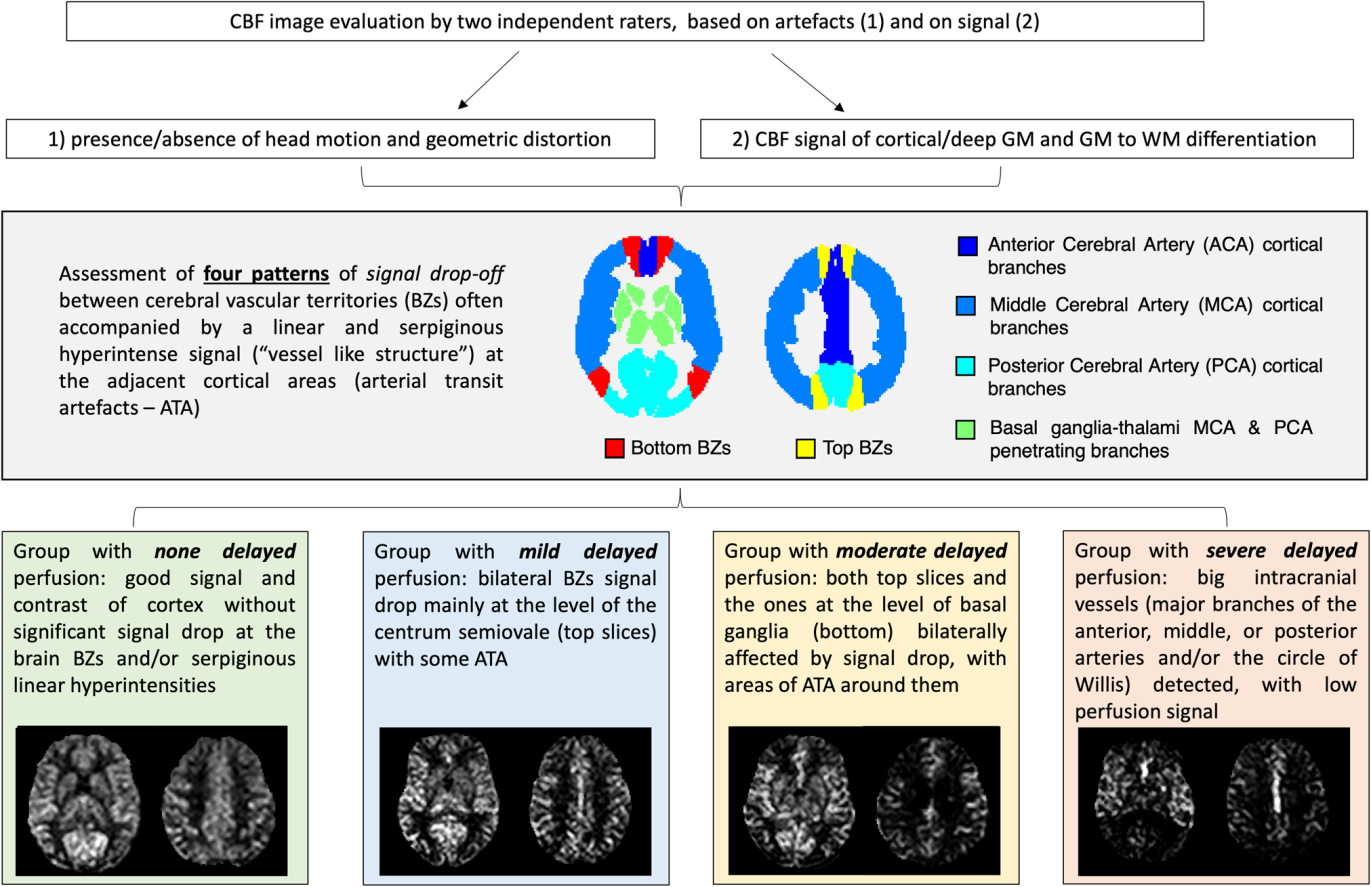
Flowchart illustrating the visual assessment of ASL maps by two independent raters. After initial quality control, a four-level visual rating scale was applied to categorize the patterns of delayed perfusion, from none to severely delayed. The schematic representation of border zones (BZs) at the level of the basal ganglia (bottom BZs, red) and of the centra semiovale (top BZs, yellow) is overlaid onto a representative vascular territories template. These zones were defined as areas in immediate proximity to the boundaries of vascular territories (two between anterior and middle cerebral arteries, and two between middle and posterior cerebral arteries at each “bottom” and “top” level). Representative individual ASL pseudo-cerebral blood flow (pCBF) maps are shown for each group

The approach of the raters can be schematized in Fig. 1. This flowchart shows that the first step of the qualitative visual assessment of CBF maps consisted of (1) recognizing any artifacts in the images (e.g., head movement and geometric distortion), (2) evaluating cortical and deep gray matter signal intensity, and gray/white matter (GM/WM) contrast. This qualitative assessment was inspired by the rating scale in [20] though not all items in the artifact-based and contrast-based components were included, and a simplified approach focusing on the usability of the data was adopted.

After this preliminary evaluation, we identified on the remaining subjects unexpected recurrent patterns of signal drop-off areas at the external BZs, often accompanied by a linear and serpiginous hyperintense signal extending into adjacent cortical areas. External BZs are usually wedge-shaped and located at the convergence of the terminal branches of the anterior, middle, and posterior cerebral arteries and are visible along all the supratentorial planes of the brain, while internal/deep BZs are more linear in shape and located between penetrating and cortical arteries. These areas of signal drop at the external BZs appeared at the visual assessment particularly pronounced and more frequent in the upper slices of the brain. Considering this caudal-cranial gradient, two representative planes were chosen for the evaluation, one lower at the level of the basal ganglia (bottom BZs) and one higher at the level of the centra semiovale (top BZs). A new visual rating scale of delayed perfusion was then proposed, with four levels representing none, mild, moderate and severe delayed perfusion, as shown in detail in Fig. 1, bottom row.

Of note, the visual appearance of the perfusion maps (i.e., areas of parenchymal signal drop and intravascular signal as suggested by serpiginous vessel-like structures) indicated that the measures quantified in our database as “CBF” could not be retained as a measure of true perfusion, even considering the assumptions of the mono-compartment ASL model for blood flow quantification [22].

For this reason, we preferred to use the term “pseudo-CBF (pCBF),” still in ml/100 g/min.

### Quantitative evaluation

To support the effectiveness of our scale, multiple quantitative measures were derived for each subject:

1. Five descriptive measures of whole-brain pCBF, namely mean, median, standard deviation (STD), maximum and normalized difference of mean and median (mean–median/median). The latter measure describes the asymmetry of data; indeed, the greater the difference between mean and median is, the more skewed the histogram appears;
2. The sCoV, proposed in [12], which was calculated as the ratio between STD and mean of whole-brain pCBF (global sCOV). In addition, two territorial sCOVs were derived, one referring to the anterior–middle cerebral arteries (sCOV_ACA+MCA_), and the other one for the posterior cerebral artery (sCOV_PCA_). To do so, the ATT-based flow territories template available in ExploreASL toolbox [24] was used, after down-sampling to the 2-mm MNI space.

Of note, the five descriptive measures were also derived separately for GM and WM, with the corresponding tissue probability maps thresholded at 0.7, along with two tissue-specific sCOVs.

### Statistical analysis

A two-way random effects model of the intraclass correlation coefficient with absolute agreement (aICC) was calculated along with Cohen’s Kappa coefficient to quantify the agreement between raters [25, 26]. Chi-square tests were applied to statistically compare the proportion of the three clinical phenotypes and the presence of cardiovascular risk factors across subjects in the four groups, to determine whether a relationship between clinical factors and delayed perfusion patterns was present.

Separate one-way ANOVAs followed by pairwise comparisons with Bonferroni-corrected post hoc tests, where appropriate, were performed to compare the five descriptive measures and sCoVs across the four delayed perfusion groups. Spearman correlation was performed to assess for associations between global sCoV and clinical/imaging variables, namely age, age-related WM changes (ARWMC), and the five descriptive measures. For all statistical tests, the significance threshold was set at *p* < 0.05 (MATLAB R2020a and SPSS Statistics v.26).

## Results

Starting from the cohort of 149 subjects recruited from a memory clinic according to the inclusion/exclusion criteria previously described in Materials and Methods, five patients were excluded after the first QC step due to the presence of gross artifacts that lowered the quality of the pCBF maps to an uninterpretable level. One hundred and forty-four subjects were thus included in the following analyses (72 ± 7 years; 76 women). Thirty-six cognitively unimpaired people had subjective cognitive decline. Thirty-seven MCIs (42%) were due to Alzheimer’s disease (AD) or mixed (AD and vascular), and 51 (58%) to other reasons. Twenty patients with dementia were due to AD (65%), three (15%) to frontotemporal degeneration, two (10%) to dementia with Lewy bodies, one (5%) to mixed degenerative and vascular causes, and one (5%) to other neurodegenerative disorders. Demographic, cognitive, and imaging variables are reported in Table 1.

### Raters and visual evaluation

Fifty-one subjects presented none (22) or mild delayed (29) perfusion, while 53 showed a clear signal drop at the top/bottom BZs and surrounding ATA (moderate group). Forty exhibited severely delayed perfusion. These results were not associated with either clinical diagnoses (MCI, dementia), cardiovascular risk factors such as hypertension, or significant cardiovascular medical history and medication, as demonstrated by the distribution of these factors in each of the four delayed perfusion groups (Table 2).

**Table 2:** Distribution of the three clinical phenotypes and presence of cardiovascular risk factors, across the four delayed perfusion groups

	None	Mild	Moderate	Severe	*p*-value
	(*n* = 22)	(*n* = 29)	(*n* = 53)	(*n* = 40)	
Cognitively unimpaired	6 (27%)	11 (38%)	11 (21%)	8 (20%)	0.296
Mild cognitive impairment	15 (68%)	15 (52%)	33 (62%)	25 (63%)	0.659
Dementia	1 (5%)	3 (10%)	9 (17%)	7 (17%)	0.428
Cardio-vascular disease* and ongoing medication	6 (27%)	11 (38%)	17 (32%)	16 (40%)	0.723
Hypertension°	7 (32%)	9 (31%)	18 (34%)	19 (48%)	0.423
Hypercholesterolemia°	9 (41%)	13 (45%)	21(40%)	24 (60%)	0.236
Smoker°	3 (14%)	2 (7%)	5 (9%)	1 (3%)	0.409

*P*-values refer to chi-square tests, while percentages are calculated for each delayed perfusion group separately

*Stenosis, stent, by-pass, TIA, myocardial infarction, coronary disease, stroke, arteritis/vasculopathy. ° Vascular risk factors

The reliability analysis of the rating scale revealed an almost perfect agreement between the visual evaluations from the two raters with Cohen’s Kappa = 0.931 and aICC = 0.989 (95CI:0.948-0.991).

**Fig. 2:**
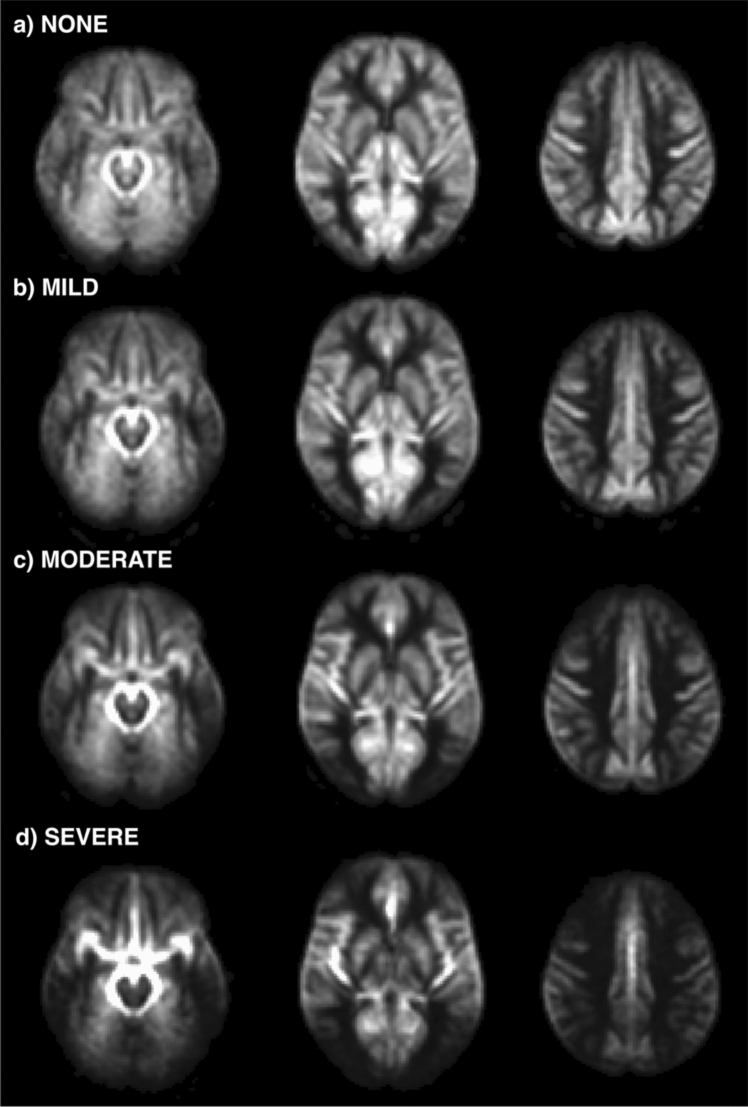
Average pseudo-cerebral blood flow (pCBF) maps, calculated across subjects belonging to the same visual delayed perfusion group. Three representative slices in the MNI space are shown and the same grayscale range is employed for visualization (0–150 ml/100 g/min). A progressive increase in cranial and caudal extension of signal drop areas as well as an increase in arterial transit artifacts at the level of the circle of Willis and its main subdivision branches are visible from mild-to-severe delayed perfusion

The average CBF maps, calculated across the subjects belonging to the same delayed perfusion group, are reported in Fig. 2, confirming the patterns detected at individual level.

### Quantitative evaluation

**Fig. 3:**
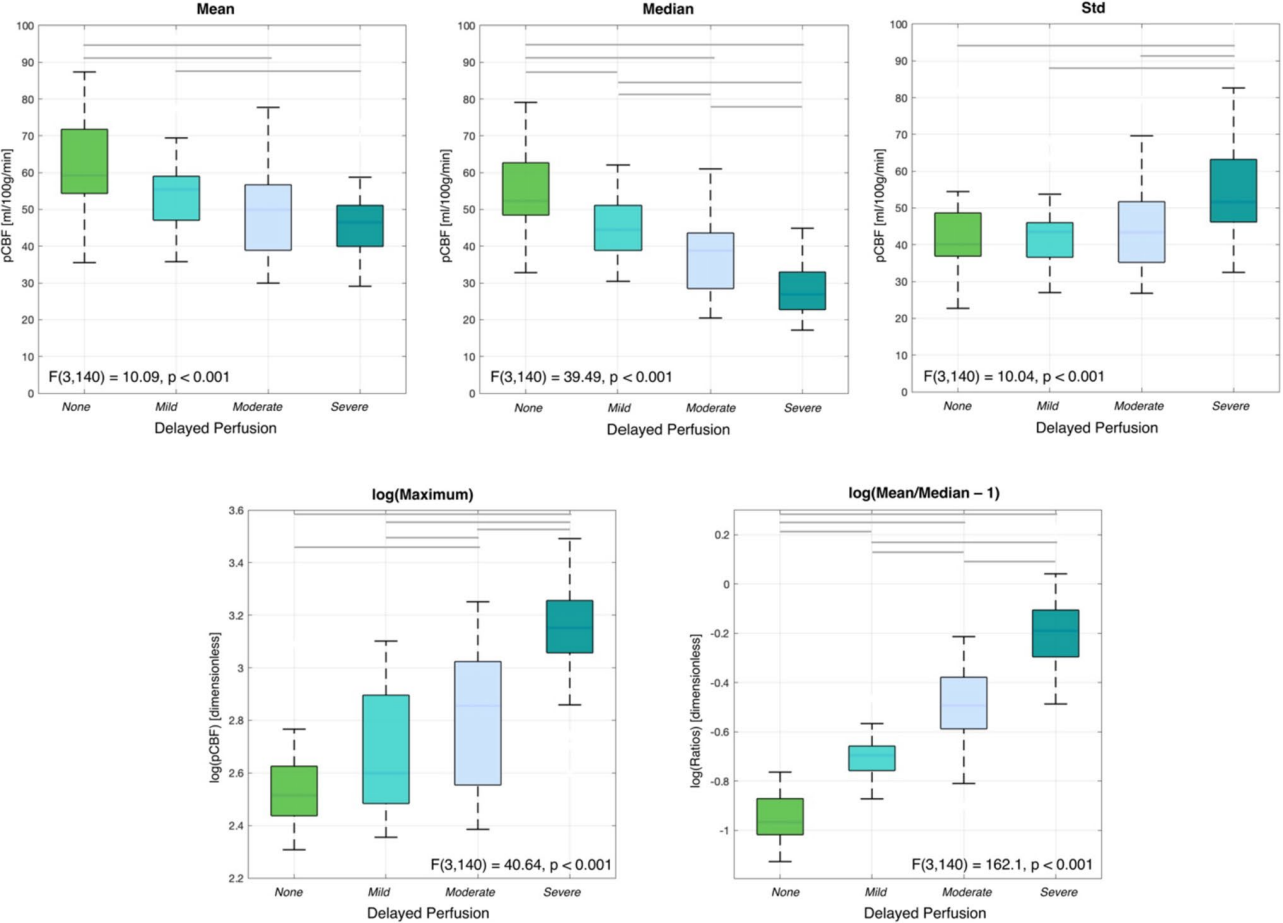
Boxplots representing the distribution of mean, median, STD, log(maximum) and log(mean/median-1) whole-brain pseudo-cerebral blood flow (pCBF) values in the four delayed perfusion groups. Boxplots denote the first and third quartiles, whiskers the min/max values excluding outliers. Overlaid gray lines represent significant post hoc tests following ANOVA analyses (*p*_Bonf_ < 0.05)

All five descriptive measures of whole-brain pCBF resulted to be associated with the visual rating classes (Fig. 3). ANOVA analyses were indeed significant for all five parameters (*p* < 0.05); however, the Bonferroni-corrected post hoc tests applied to each of these ANOVAs yielded different results, as highlighted in Fig. 3. Mean and STD perfusion values proved to be the more stable indices across the four delayed perfusion groups from the visual scale, with only few significant differences in post hoc analysis. Notably, for STD, only comparisons with the severe group were statistically significant (*p*_Bonf_ < 0.05), while in all other cases no significant results were found. Conversely, median values were significantly reduced across the scale. All post hoc comparisons were significant, (*p*_Bonf_ < 0.05). Log-transformed maximum pCBF values were significantly increasing along with the visual scale (except for none vs mild), and the same pattern was found for log-transformed normalized mean/median difference, with all post hoc comparisons being significant (*p*_Bonf_ < 0.05). Highly similar patterns were found when considering CBF measures for GM and WM (Supplementary Fig. S1–S2).

**Fig. 4:**
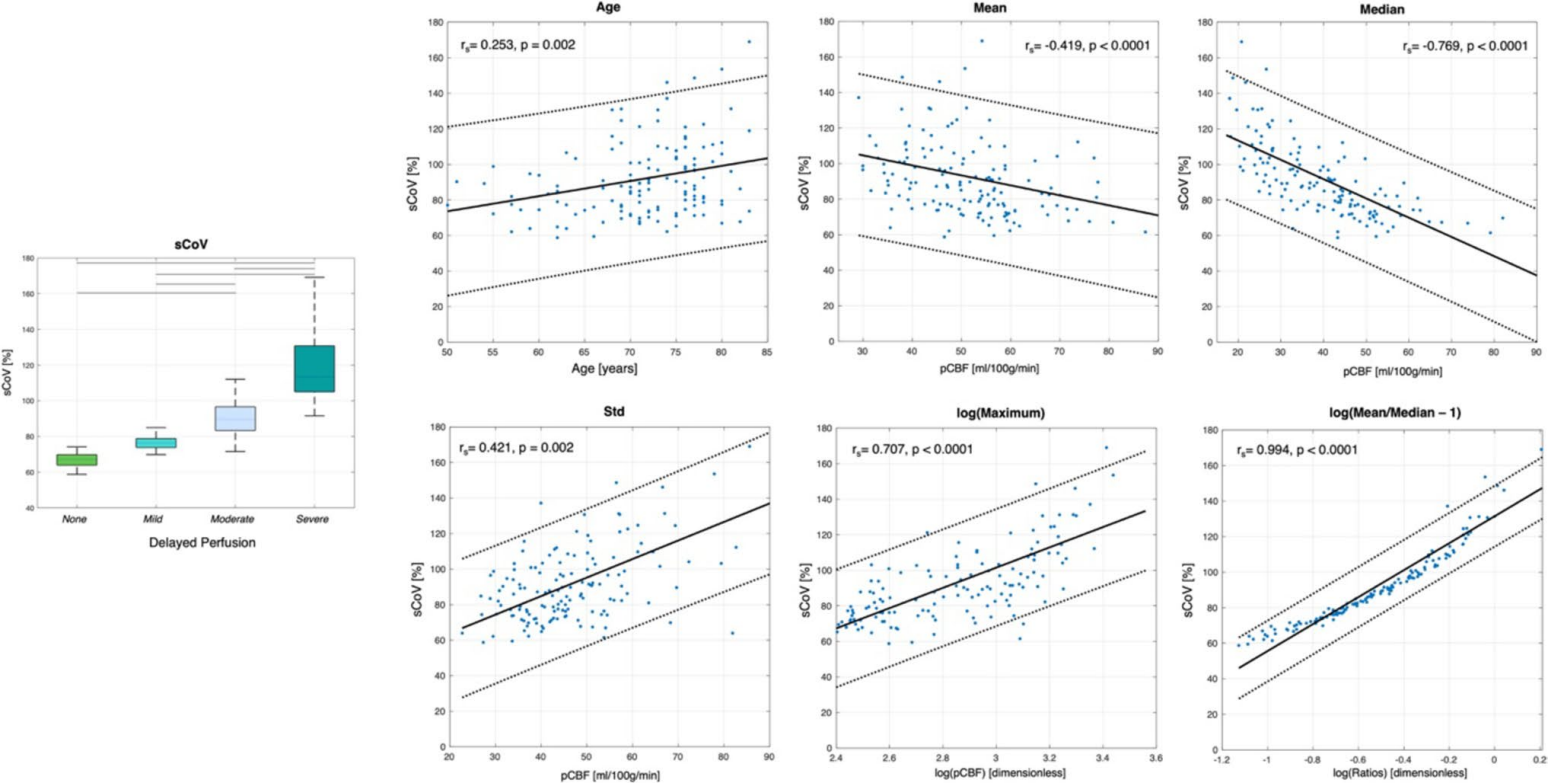
Boxplots of global spatial CoV values (sCoV, %) for the four delayed perfusion groups, with gray lines representing significant post hoc tests following ANOVA (*p*_Bonf_ < 0.05). The significant Spearman correlations between global sCoV and clinical/imaging variables are also reported

The distribution of global sCoV values is reported for each of the four delayed perfusion groups in Fig. 4, highlighting an association between this ATT proxy and the visual scale, with significantly increased values when delayed perfusion was increasingly found in visual assessment (*F*(3,140) = 89.45, *p* = 2.21e − 32; all post hoc tests were significant at *p*_Bonf_ < 0.05, except for none vs mild with p_Bonf_ > 0.05). These differences were confirmed when considering the two tissue-specific (sCOV_GM_, sCOV_WM_) and territory-specific (sCOV_ACA+PCA_, sCOV_MCA_) sCOV measures (*p*_Bonf_ < 0.05, Supplementary Figs. S3–S4). Of note, in the latter case, within-group significant differences were found for each of the four groups in the visual QC scale, with higher sCOV values for PCA compared to ACA + MCA (paired *t*-tests, *p*_Bonf_ < 0.05).

The correlation of global sCoV with age and the five descriptive parameters revealed significant associations in all cases (*p* ≤ 0.002, highest for the normalized mean/median difference, Fig. 4). Conversely, the correlation with ARWMC did not reach significance (*p* = 0.369).

Finally, global sCoV values did not significantly differ across the three clinical phenotypes (*F*(2,141) = 1.06, *p* = 0.35; controls: 87.6 ± 15.8%, MCI: 93.7 ± 27.1%, dementia: 95.4 ± 19.8%).

## Discussion

The main objective of our study was how to assess the hemodynamic information hidden in single-timepoint ASL maps of older subjects, by proposing a practical visual scale and by comparing it with measured sCoV. According to the visual assessment of artifact and contrast-based components of the recurrent patterns, our results demonstrated that the quality of single-timepoint CBF maps can be biased by ATT delays in MCI and dementia patients as well as healthy older subjects, with 85% of them having mild (20%), moderate (37%) and severe (28%) delayed perfusion as identified by the visual scale. This observation could not be explained by clinical diagnoses, as the number of MCI and dementia patients across the four delayed perfusion groups did not significantly differ, and no association was found with known cardiovascular disease, as retrieved from clinical data (hypertension, hyperlipidemia, smoking) and pharmacotherapy, as opposed to results reported in [17], where higher sCOV was associated with cardiovascular risk factors and history of heart disease. This difference could relate to inter-individual physiological/pathological variability and age-related cardiovascular changes [27], but also to the ASL sequence type, as the longer bolus duration in pseudo-continuous ASL (pCASL) might reduce pCBF sensitivity to ATT changes [28].

In the current literature, single-timepoint ASL is usually reported as being sufficient for flow estimation in nonvascular disease [8, 9], and consequently, it is widely used, considering also it is faster, less prone to movement artifacts, and associated with higher SNR than multi-timepoint. Our results could thus lead to increased awareness in ASL sequence selection, suggesting that if the target is a precise CBF measurement, this can only be achieved by multi-timepoint approaches [29]. Indeed, the observed influence of blood delay argues in favor of the use of multi-timepoint ASL whenever it is important to obtain accurate and reliable CBF quantification, which is also essential for comparing ASL results with other perfusion and metabolic imaging modalities. Furthermore, our results suggest that a visual assessment of perfusion maps should be performed before proceeding to any subsequent analyses, as further confirmed by the marked changes depicted across the pCBF distribution statistics in the four delayed perfusion groups. The practical QC workflow we propose includes two different levels. The *first one* is the recognition of any gross artifacts related to motion or geometric distortion that could affect image quality, and the evaluation of signal quality/contrast. Though inspired by the comprehensive score system proposed in [20], we here present a simpler approach that does not require scoring multiple images and specific items but aims at easily identifying highly corrupted CBF maps that should be discarded in any setting. The *second* level of the QC scale is represented by our new four-level visual scale that focuses specifically on rating ATT patterns, adding to the first level of global QC the assessment of the signal drop/ATA at the top and bottom BZs. This score is based on the fact that the signal drop between the different watershed regions of the semioval centers and the basal nuclei is recognizable on both sides and from front to back, which makes the scale susceptible to ATT delays due to inter-individual physiological/pathological variability and age-related cardiovascular changes [27] and less so to those attributable to regional pathologies (e.g., unilateral carotid stenosis).

Our systematic approach showed robustness between raters, suggesting its usability and reproducibility in clinical routine. Moreover, both raters felt it to be a useful and easy-to-use system that provides important indications on image quality and cerebrovascular integrity, in any clinical or research settings. Importantly, this scale can be applied to any ASL sequence, not only to 3D FAIR ASL data.

If the accuracy of CBF estimation is a limitation of single-timepoint sequences, sCoV allows to obtain additional quantitative hemodynamic information, which has been shown to be strongly related to ATT measures from multi-timepoint sequences [12]. SCoV has been used in several studies already [7, 13–17], revealing an association between sex, age, and hypertension in a population of older patients, and providing a useful metric to assess hemodynamic impairment in moyamoya disease [13], though dependent on the chosen post-labeling delay. It is sensitive to varying degrees of cognitive change in MCI and early dementia and, more generally, able to distinguish cognitively unimpaired from cognitively impaired individuals [7, 16, 17].

Previous studies on cognitive impairment and dementia using an ATT surrogate marker found inconsistent results, with some authors showing moderate evidence for an increasing sCoV in total GM across the AD trajectory [7], while others only described an increased value between cognitively unimpaired and MCI patients, and not between MCI and AD patients [15]. Results from previous studies measuring ATT directly in AD are also discrepant, showing no difference in the abovementioned parameters when using a case–control paradigm [30], while a significant ATT prolongation was described by other authors in AD when compared to controls [31], raising the assumption of a component of underlying vascular impairment. In the here investigated cohort, no significant differences were depicted by the sCoV across the three clinical groups, suggesting that severe hemodynamic delays may be present even in normally functioning older subjects, without being associated with cognitive impairment or dementia conditions.

The lack of significant differences across our clinical groups is not surprising and confirms, on one hand, that evaluation for hemodynamic impairment is warranted in all subjects, as it might be encountered in cognitive unimpaired individuals with different degrees of severity, and on the other it suggests that the high variability usually observed when comparing MCI and dementia patients also reflects different delayed perfusion patterns, confirming an intricate relationship between neurodegeneration and cerebrovascular health. Future studies aiming at specifically studying ATT delays in neurodegenerative disorders with different ASL sequences and higher size of each clinical group should rely on strict inclusion criteria to ensure that only similar patients are included, in order to disentangle purely disease-related hemodynamic impairment. Finally, our study also highlighted in all subjects and groups significantly different sCOV measures when comparing the anterior/middle and posterior circulation territories. This suggests that prolonged ATTs are present in the posterior circulation, even when subjects were classified as having none delayed perfusion at the visual scale. This is in line with previous studies with multi-timepoint sequences, showing longer ATTs in the posterior compared to the anterior circulation, and could further aid the visual interpretation of the ASL-derived maps, especially when dealing with patient data.

The main limitation of our study is the type of single-timepoint ASL sequence adopted (3D PASL) that can be more prone to influences of ATT than pCASL which provides a more robust signal [9]. However, this intrinsic weakness can also be seen as an advantage because it has initiated our quest to unveil hemodynamic information hidden in the 3D PASL sequence and, possibly, in other single-timepoint schemes. The use of a single TI (2000 ms) is also a limitation, but being the standard parameter setting of the commercial sequence that was adopted, it represents the clinical reality encountered in many institutions. When interpreting 3D PASL images, it is essential to raise a note of caution and take this into consideration when reporting findings, considering the impact it may have on clinicians’ practice. Further investigations using different labeling schemes (pCASL), longer TIs (> 2000 ms) and readouts (2D) are needed to provide a wider picture of CBF patterns from single-timepoint ASL sequences.

## Conclusions

The advantages of using ASL MRI in clinics are undeniable, but our study confirms that single-timepoint ASL data are severely affected by delayed ATT and cannot be used reliably in either clinical or research settings. Future recommendations for the use of ASL could change current practice, favoring greater use of multi-timepoint sequences or maintaining the use of single-timepoint but supplementing it with visual scale assessments and/or sCoV measures.

### Supplementary Information

Below is the link to the electronic supplementary material.

Supplementary file1 (PDF 991 KB)

## Abbreviations

ASL: Arterial spin labeling
CBF: Cerebral blood flow
sCoV: Spatial coefficient of variation
MCI: Mild cognitive impairment
ATT: Arterial transit time
ATA: Arterial transit artifacts
BZs: Border zones
QUASAR: Quantitative STAR labeling of arterial regions
QC: Quality control
SNR: Signal-to-noise ratio
GM: Gray matter
WM: White matter
ARWMC: Age-related white matter changes

## References

[1] Wolf RL, Detre JA (2007). Clinical neuroimaging using arterial spin-labeled perfusion magnetic resonance imaging. Neurotherapeutics.

[2] Camargo A, Wang Z (2021). Longitudinal cerebral blood flow changes in normal aging and the Alzheimer's disease continuum identified by arterial spin labeling MRI. JAD.

[3] Lindner T, Bolar D, Achten E et al (2023) Current state and guidance on arterial spin labeling perfusion MRI in clinical neuroimaging. Magn Reson Med (in press). 10.1002/mrm.29572. Online ahead of print. 10.1002/mrm.29572 PMC10914350 36695294

[4] Musiek ES, Chen Y, Korczykowski M (2012). Direct comparison of fluorodeoxyglucose positron emission tomography and arterial spin labeling magnetic resonance imaging in Alzheimer’s disease. Alzheimers Dement.

[5] Chen Y, Wolk DA, Reddin JS (2011). Voxel-level comparison of arterial spin-labeled perfusion MRI and FDG-PET in Alzheimer disease. Neurology.

[6] Van Osch MJP, Teeuwisse WM, Chen Z (2018). Advances in arterial spin labelling MRI methods for measuring perfusion and collateral flow. J Cerebral Blood Flow Metabolism.

[7] Morgan C, Melzer T, Roberts RP (2021). Spatial variation of perfusion MRI reflects cognitive decline in mild cognitive impairment and early dementia. Sci Rep.

[8] Haller S, Zaharchuk G, Thomas DL (2016). Arterial spin labeling perfusion of the brain: emerging clinical applications. Radiology.

[9] Alsop DC, Detre JA, Golay X (2015). Recommended implementation of arterial spin-labeled perfusion MRI for clinical applications: a consensus of the ISMRM perfusion study group and the European consortium for ASL in dementia. MRM.

[10] Zaharchuk G, Bammer R, Straka M (2009). Arterial spin-label imaging in patients with normal bolus perfusion-weighted MR imaging findings: pilot identification of the borderzone sign. Radiology.

[11] Hendrikse J, Petersen ET, van Laar PJ (2008). Cerebral border zones between distal end branches of intracranial arteries: MR imaging. Radiology.

[12] Mutsaerts HJMM, Petr J, Vaclavu L (2017). The spatial coefficient of variation in arterial spin labeling cerebral blood flow images. J Cerebral Blood Flow Metabolism.

[13] Hara S, Tanaka Y, Inaji M (2022). Spatial coefficient of variation of arterial spin labeling MRI for detecting hemodynamic disturbances measured with 15 O-gas PET in patients with moyamoya disease. Neuroradiology.

[14] Ibaraki M, Nakamura K, Toyoshima H (2019). Spatial coefficient of variation in pseudo-continuous arterial spin labeling cerebral blood flow images as a hemodynamic measure for cerebrovascular steno-occlusive disease: a comparative 15O positron emission tomography study. J Cereb Blood Flow Metab.

[15] Hafdi M, Mutsaerts HJ, Petr J, Richard E, van Dalen JW (2022) Atherosclerotic risk is associated with cerebral perfusion–a cross-sectional study using arterial spin labeling MRI. NeuroImage: Clinical 36:103142 10.1016/j.nicl.2022.103142 PMC9400119 35970112

[16] Shirzadi Z, Stefanovic B, Mutsaerts HJMM (2019). Classifying cognitive impairment based on the spatial heterogeneity of cerebral blood flow images. J Magn Reson Imaging.

[17] Gyanwali B, Tan CS, Petr J (2022). Arterial spin-labeling parameters and their associations with risk factors, cerebral small-vessel disease, and etiologic subtypes of cognitive impairment and dementia. Am J Neuroradiol.

[18] Lee T, Fischbein NJ, André JB (2012). Identification of venous signal on arterial spin labeling improves diagnosis of dural arteriovenous fistulas and small arteriovenous malformations. AJNR Am J Neuroradiol.

[19] Yoo RE, Yun TJ, Rim JH (2015). Bright vessel appearance on arterial spin labeling MRI for localizing arterial occlusion in acute ischemic stroke. Stroke.

[20] Fallatah SM, Pizzini FB, Gomez-Anson B (2018). A visual quality control scale for clinical arterial spin labeling images. Eur Radiol Exp.

[21] Ribaldi F, Chicherio C, Altomare D, Martins M, Tomczyk S, Jelescu I, Maturana E, Scheffler M, Haller S, Lövblad KO, Pievani M, Garibotto V, Kliegel M, Frisoni GB (2021). Brain connectivity and metacognition in persons with subjective cognitive decline (COSCODE): rationale and study design. Alzheimers Res Ther.

[22] Buxton R, Frank L, Wong E, et al (1998) A general kinetic model for quantitative perfusion imaging with arterial spin labeling. MRM, pp 383–396. 10.1002/mrm.1910400308 10.1002/mrm.1910400308 9727941

[23] Groves A, Chappell MA, Woolrich M (2009). Combined spatial and non-spatial prior for inference on MRI time-series. Neuroimage.

[24] Mutsaerts HJMM, Petr J, Groot P (2020). ExploreASL: an image processing pipeline for multi-center ASL perfusion MRI studies. Neuroimage.

[25] Koo TK, Li MY (2016). A guideline of selecting and reporting intraclass correlation coefficients for reliability research. J Chiropr Med.

[26] McHugh M (2012). Interrater reliability: the kappa statistic. Biochem Med.

[27] Roberts GS, Peret A, Jonaitis EM (2023). Normative cerebral hemodynamics in middle-aged and older adults using 4D Flow MRI: initial analysis of vascular aging. Radiology.

[28] Dai W, Fong T, Jones RN (2017). Effects of arterial transit delay on cerebral blood flow quantification using arterial spin labeling in an elderly cohort. J Magn Reson Imaging.

[29] MacIntosh BJ, Lindsay AC, Kylintireas I (2010). Multiple inflow pulsed arterial spin-labeling reveals delays in the arterial arrival time in minor stroke and transient ischemic attack. AJNR Am J Neuroradiol.

[30] Yoshiura T, Hiwatashi A, Noguchi T (2009). Arterial spin labelling at 3-T MR imaging for detection of individuals with Alzheimer’s disease. Eur Radiol.

[31] Mak H, Chan Q, Zhang Z (2012). Quantitative assessment of cerebral hemodynamic parameters by QUASAR arterial spin labeling in Alzheimer's disease and cognitively normal elderly adults at 3-tesla. JAD.

