# Supplementary material for: Insights into single-timepoint ASL hemodynamics: what visual assessment and spatial coefficient of variation can tell
Source: Radiol Med. 2024 Feb 8;129(3):467–77. doi: 10.1007/s11547-024-01777-z (PMC10943156; doi:10.1007/s11547-024-01777-z)
Supplement: Supplementary file 1 — Supplementary file1 (PDF 991 KB) [file 11547_2024_1777_MOESM1_ESM.pdf]

Insights into single-timepoint ASL hemodynamics: what visual assessment and spatial coefficient of variation can tell

Francesca Benedetta Pizzini, Ilaria Boscolo Galazzo, Valerio Natale, Federica Ribaldi, Max Scheffler, Ferdinando Caranci, Karl-Olof Lovblad, Gloria Menegaz, Giovanni Battista Frisoni, Matthias Gunther

Corresponding author:
Francesca Benedetta Pizzini
Department of Engineering for Innovation Medicine
University of Verona
Piazzale L.A. Scuro, 10, 37100, Verona, Italy
Phone: +39 045 802 4301
Email: francescabenedetta.pizzini@univr.it

Supplementary Figure S1.

Boxplots representing the distribution of mean, median, std, log(maximum) and log(mean/median-1) gray matter pseudo-cerebral blood flow (pCBF) values in the four delayed perfusion groups. Boxplots denote the first and third quartiles, the shaded line the median, whiskers the min/max values excluding outliers. Overlaid gray lines represent significant post-hoc tests following ANOVA analyses ($p_{Bonf} < 0.05$).

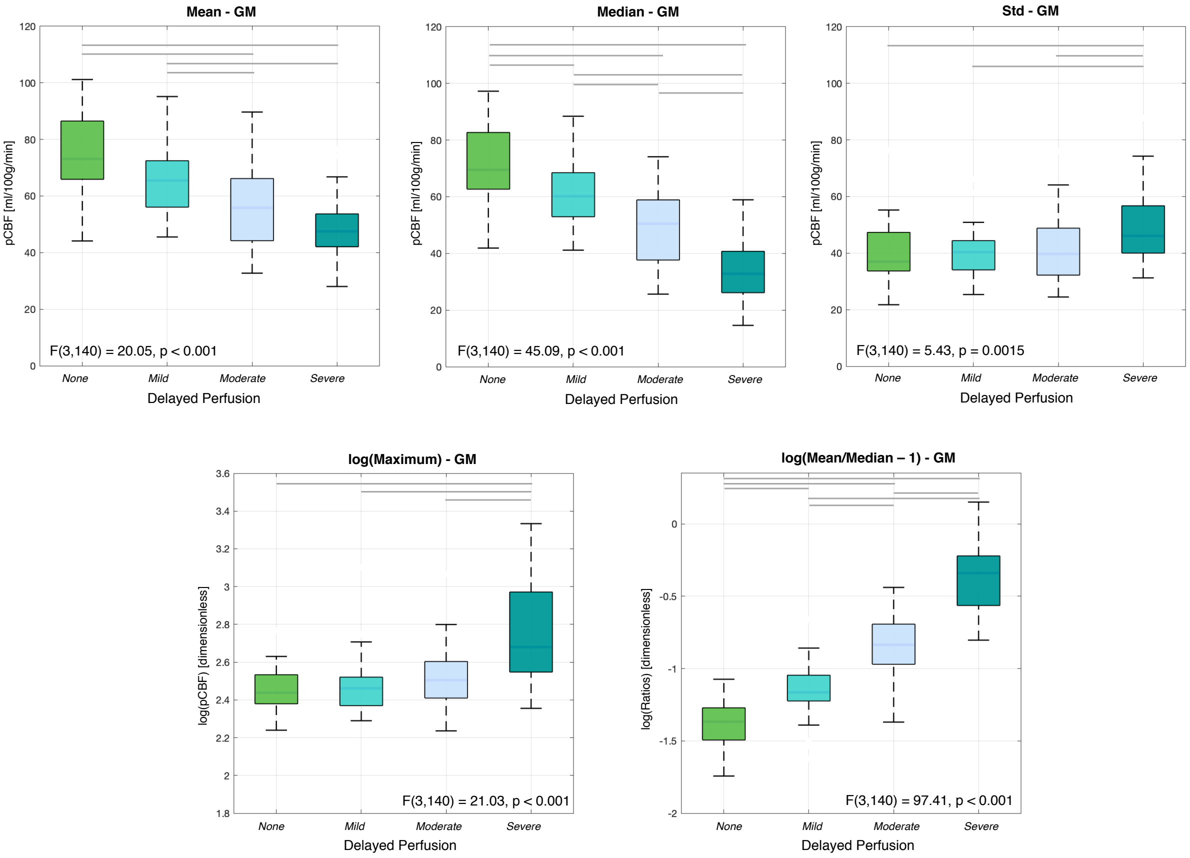

Supplementary Figure S2. Boxplots representing the distribution of mean, median, std, log(maximum) and log(mean/median-1) white matter pseudo-cerebral blood flow (pCBF) values in the four delayed perfusion groups. Overlaid gray lines represent significant post-hoc tests following ANOVA analyses ($p_{Bonf} < 0.05$). For interpretation of the boxplots please refer to Figure S1.

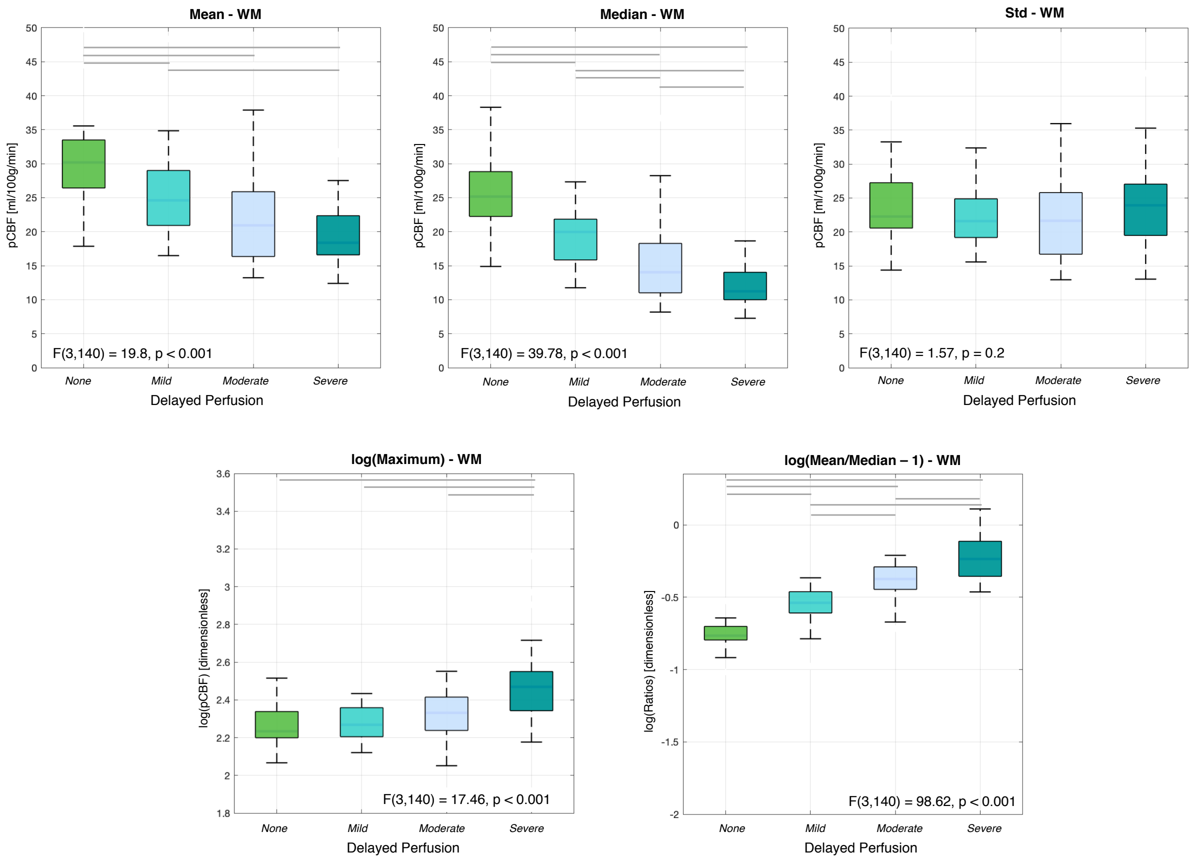

Supplementary Figure S3. Boxplots of spatial CoV values (sCOV, %) for the two tissue-specific measures, calculated for gray and white matter tissues (GM/WM) in the four delayed perfusion groups. Gray lines represent statistically significant post-hoc tests following ANOVA (p_{Bonf}<0.05).

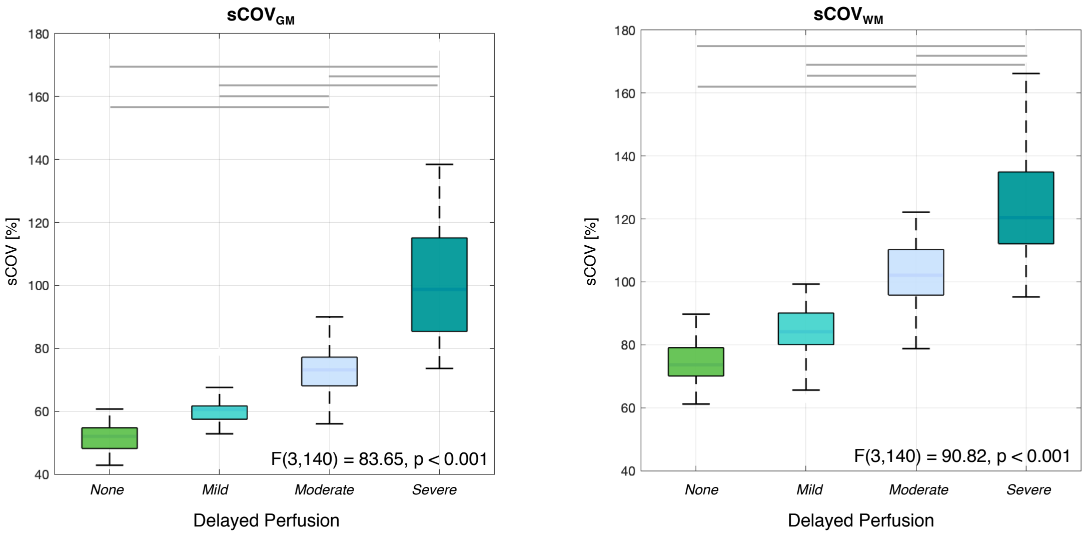

Supplementary Figure S4. Boxplots of spatial CoV values (sCOV, %) for the two territorial-specific measures, one related to the anterior/middle circulation territories ($sCOV_{ACA+MCA}$) and the other one for the posterior circulation territories ($sCOV_{PCA}$) for the four delayed perfusion groups. Gray lines represent statistically significant post-hoc tests following ANOVA ($p_{Bonf}<0.05$).

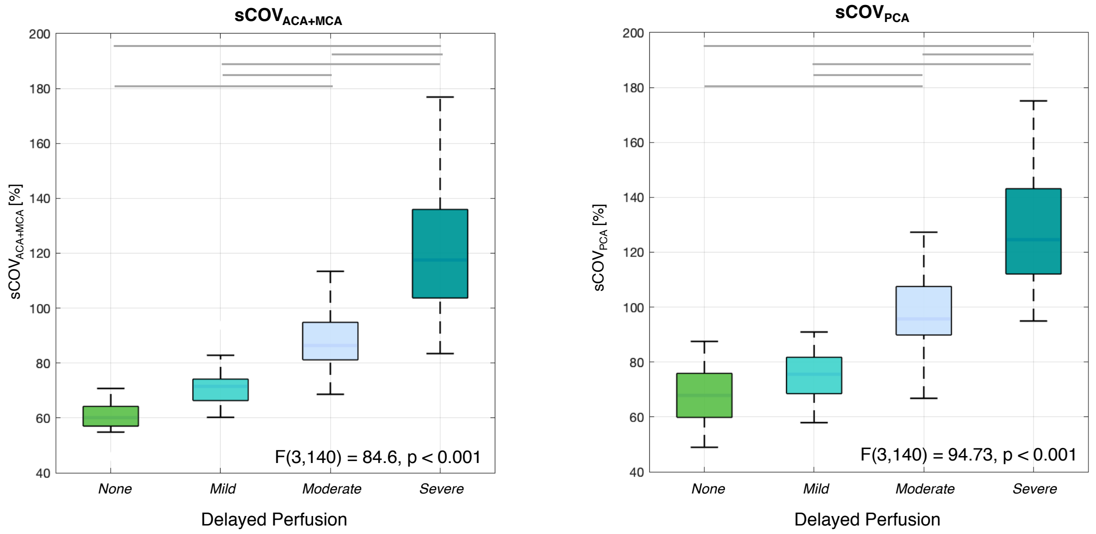

Supplementary Table S1. Mean ± standard deviation (std) pseudo-cerebral blood flow (pCBF) values calculated across subjects belonging to the same delayed perfusion group (none, mild, moderate, severe) for the five descriptive measures (mean, median, std, log(maximum) and log(mean/median-1)). Separate values for whole brain, gray and white matter are reported. Mean, median and std are expressed as ml/100g/min, while the other two measures are dimensionless.

	Mean			Median			Std			log(Max)			log(Mean/Median - 1)		
	**WB**	**GM**	**WM**	**WB**	**GM**	**WM**	**WB**	**GM**	**WM**	**WB**	**GM**	**WM**	**WB**	**GM**	**WM**
None	63.8 ± 18.5	77.4 ± 22.5	32.7 ± 10.2	57.1 ± 16.9	74.2 ± 21.8	27.9 ± 8.9	42.5 ± 12.2	40.2 ± 11.9	24.3 ± 7.7	2.5 ± 0.2	2.4 ± 0.1	2.2 ± 0.1	-0.9 ± 0.2	-1.5 ± 0.1	-0.7 ± 0.1
Mild	55.4 ± 11.9	66.5 ± 14.2	25.8 ± 6.3	46.1 ± 10.1	61.9 ± 13.4	20.2 ± 6.1	43.1 ± 8.9	40.1 ± 8.7	22.4 ± 4.4	2.67 ± 0.2	2.5 ± 0.2	2.3 ± 0.1	-0.7 ± 0.1	-1.2 ± 0.2	-0.5 ± 0.1
Moderate	49.6 ± 12.4	56.6 ± 14.4	21.8 ± 6.8	37.5 ± 10.2	49.1 ± 13.1	15.4 ± 5.6	44.3 ± 11.2	41.3 ± 10.5	22.0 ± 5.9	2.8 ± 0.2	2.5 ± 0.1	2.3 ± 0.1	-0.5 ± 0.2	-0.8 ± 0.2	-0.4 ± 0.1
Severe	46.6 ± 9.2	48.5 ± 10.4	19.4 ± 4.7	27.8 ± 7.1	34.0 ± 10.0	12.0 ± 3.0	56.2 ± 15.8	49.2 ± 12.7	24.6 ± 8.2	3.1 ± 0.2	2.7 ± 0.3	2.5 ± 0.2	-0.2 ± 0.2	-0.4 ± 0.2	-0.2 ± 0.1

WB = whole brain; GM = grey matter; WM = white matter

Supplementary Table S2. Mean ± standard deviation (std) values for the different spatial CoV measures (sCOV, %), that is global, territory-specific and tissue-specific, calculated across subjects belonging to the same delayed perfusion group (none, mild, moderate, severe).

	Global	Territories		Tissues	
	**sCOV**	**$sCOV_{ACA+MCA}$**	**$sCOV_{PCA}$**	**$sCOV_{GM}$**	**$sCOV_{WM}$**
None	66.7 ± 4.2	60.2 ± 5.1	67.9 ± 10.1	51.9 ± 5.1	74.5 ± 6.3
Mild	76.7 ± 4.3	71.3 ± 6.9	75.8 ± 8.5	60.2 ± 4.9	86.9 ± 8.3
Moderate	89.7 ± 8.9	87.6 ± 10.3	98.3 ± 13.5	73.6 ± 7.9	102.3 ± 9.7
Severe	120.6 ± 24.3	123.6 ± 28.7	130.3 ± 24.7	102.9 ± 24.3	125.2 ± 20.6
